# Predictive Feedback Can Account for Biphasic Responses in the Lateral Geniculate Nucleus

**DOI:** 10.1371/journal.pcbi.1000373

**Published:** 2009-05-01

**Authors:** Janneke F. M. Jehee, Dana H. Ballard

**Affiliations:** 1 Center for Visual Science and Department of Computer Science, University of Rochester, Rochester, New York, United States of America; 2 Department of Psychology, Vanderbilt University, Nashville, Tennessee, United States of America; 3 Department of Computer Science, University of Texas at Austin, Austin, Texas, United States of America; University College London, United Kingdom

## Abstract

Biphasic neural response properties, where the optimal stimulus for driving a neural response changes from one stimulus pattern to the opposite stimulus pattern over short periods of time, have been described in several visual areas, including lateral geniculate nucleus (LGN), primary visual cortex (V1), and middle temporal area (MT). We describe a hierarchical model of predictive coding and simulations that capture these temporal variations in neuronal response properties. We focus on the LGN-V1 circuit and find that after training on natural images the model exhibits the brain's LGN-V1 connectivity structure, in which the structure of V1 receptive fields is linked to the spatial alignment and properties of center-surround cells in the LGN. In addition, the spatio-temporal response profile of LGN model neurons is biphasic in structure, resembling the biphasic response structure of neurons in cat LGN. Moreover, the model displays a specific pattern of influence of feedback, where LGN receptive fields that are aligned over a simple cell receptive field zone of the same polarity decrease their responses while neurons of opposite polarity increase their responses with feedback. This phase-reversed pattern of influence was recently observed in neurophysiology. These results corroborate the idea that predictive feedback is a general coding strategy in the brain.

## Author Summary

For many neurons in the early visual brain the optimal stimulation for driving a response changes from one stimulus pattern to the opposite stimulus pattern over short periods of time. For example, many neurons in the lateral geniculate nucleus (LGN) respond to a bright stimulus initially but prefer a dark stimulus only 20 milliseconds later in time, and similar changes in response preference have been found for neurons in other areas. What would be the computational reason for these biphasic response dynamics? We describe a hierarchical model of predictive coding that explains these response properties. In the model, higher-level neurons attempt to predict their lower-level input, while lower-level neurons signal the difference between actual input and the higher-level predictions. In our simulations we focus on the LGN and area V1 and find that after training on natural images the layout of model connections resembles the brain's LGN-V1 connectivity structure. In addition, the responses of model LGN neurons are biphasic in time, resembling biphasic responses as found in neurophysiology. Moreover, the model displays a specific pattern of influence of feedback from higher-level areas that was recently observed in neurophysiology. These results corroborate the idea that predictive feedback is a general coding strategy in the brain.

## Introduction

Cells in the LGN exhibit striking receptive field dynamics. Besides their well-known center-surround organization, LGN receptive fields are characterized by bright-excitatory (dark-excitatory) regions that become dark-excitatory (bright-excitatory) over time intervals that may be as short as 20 milliseconds [1]–[3]. Biphasic responses have been described not only in the LGN, but seem to be characteristic of neurons in many visual areas. For example, biphasic responses have been observed in primary visual cortex [3],[4], and also in MT, where the optimal stimulation changes from one direction of motion to a 180° reversal in motion preference with time [5],[6]. What computational reason would neurons have to change their preferred stimulus over such short periods of time? Here, we argue that biphasic dynamics naturally follow from neural mechanisms of predictive coding.

A longstanding approach to understanding early-level processing has been to consider it in terms of efficient coding of natural images [7]–[10]. Natural images are typically highly correlated in both space and time, and a neural code that ignores these correlations would be very inefficient. It has therefore been postulated that early-level visual processing removes correlations in the input, resulting in a more sparse and statistically independent output.

Building along these lines, it has been suggested that early visual areas remove correlations by removing the predictable, and hence redundant, components in their input. For example, the center-surround structure of LGN receptive fields can be explained using predictive coding mechanisms [11],[12]. Because a center pixel intensity value in natural images can often be predicted from its surrounding values, its value can be replaced with the difference between the center value and a prediction from a linear weighted sum of its surrounding values. This decorrelates the neuronal input and removes redundancy in the outputs [7],[13].

Predictive coding may have further value as a general principle that works through interactions between all lower-order and higher-order visual areas [14]–[16]. Low-order and high-order visual areas are reciprocally connected [17], and responses of neurons in these areas are often correlated due to their overlapping receptive fields. To reduce redundancy and decorrelate the visual responses, low-level visual input could therefore be replaced by the difference between the input and a prediction from higher-level structures. Put another way, higher-level receptive fields could represent the predictions of the visual world, while lower-level areas could signal the error between predictions and the actual visual input [14]–[16],[18],[19]. An advantage of feedback interactions over local, within-area computations is that higher-level cortical receptive fields are larger and encode more complex stimuli, therefore allowing for complex predictions about large portions of the visual field. This hypothesis has been shown to account for steady state extra-classical receptive field effects such as end-stopping [14].

Here, we show by simulation that biphasic responses may result from similar interactions with higher-order areas, which remove redundancy by removing the predictable components in their input. We focus on the LGN and V1, for which the feedforward-feedback connectivity structure and bottom-up inputs are fairly well-known. Although responses of LGN cells tend to follow many of the characteristics of their retinal input [20], biphasic responses are stronger in geniculate neurons than in the retinal neurons driving their response [21]. We show that these stronger rebound effects in LGN may result from predictive feedback interactions with area V1. Moreover, after training on natural images, the model exhibits the brain's LGN-V1 connectivity structure, and it displays a phase-reversed pattern of influence of feedback on LGN cells. This phase-reversed pattern of influence was recently observed in neurophysiology [22].

## Results

### Hierarchical model of predictive coding

**Figure 1:**
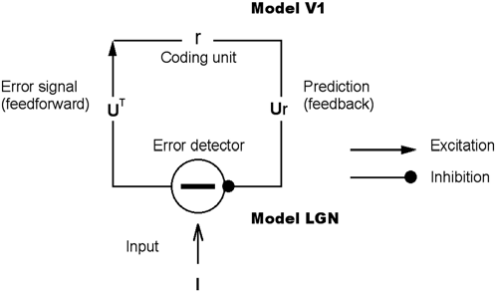
Hierarchical model for predictive coding. Higher-level coding units attempt to predict the responses of units in the next lower level via feedback connections, while lower-level error detectors signal the difference between the prediction and the actual input. Feedforward connections encode the synaptic weights represented by a matrix 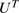. Higher-level units maintain the current estimate of the input signal 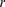 and convey the top-down prediction 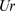 to the lower level via feedback connections. Difference detectors compute the difference 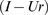 between current activity 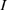 and the top-down prediction 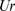.

The model consists of two layers (Fig. 1). The first layer, which corresponds to part of the lateral geniculate nucleus, consists of on-center and off-center type units, with on-center type units coding for brighter stimulus regions and off-center type units coding for darker regions. The model's next higher level, which corresponds to an orientation column in primary visual cortex, receives input from the model LGN through feedforward connections. After receiving its LGN input, the feedforward V1 receptive field that best matches the input (i.e. the one that makes the most likely prediction) is selected with high probability, and the selected neuron feeds its prediction back to model LGN. The layout of feedback connections follows the structure of feedforward connections, as has been found experimentally [22],[23]. LGN neurons then compute the error between the higher-level prediction and the actual input. This error is sent forward to correct the higher-level prediction, and the entire process is repeated in the next feedforward-feedback cycle. Thus, a feedforward-feedback cycle comprises lower-level error detectors correcting higher-level predictions, and higher-level responses updating the lower-level error signals, similar to some previous models [14],[16],[18],[19],[24]. We assume that a single feedforward-feedback cycle takes around 20 milliseconds, but our results do not critically depend on the value of this parameter (see Methods).

Connection weights of the model are adapted to the input by minimizing the description length or entropy of the joint distribution of inputs and neural responses (Methods, see also [25]). This minimizes the model's prediction errors and improves the sparseness of the neural code. Thus, for any given input, the model converges to a set of connection weights that is optimal for predicting that input. The model is trained on image patches extracted from natural scenes, as receptive field properties might be largely determined by the statistics of their natural input [7],[11],[14],[26].

### LGN-V1 connectivity structure after training

**Figure 2:**
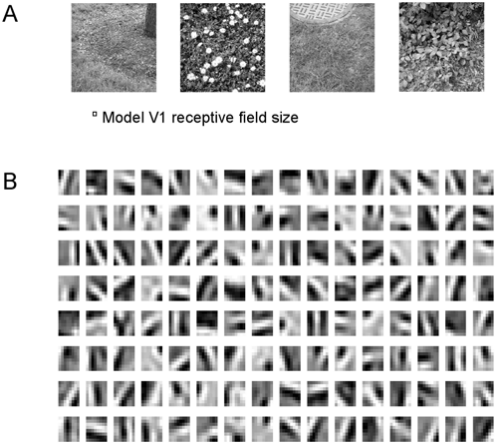
Receptive fields of model V1 units after training on natural images. (A) Examples of natural images used for training. The square denotes model V1 receptive field size. (B) V1 receptive fields after training. Plots are scaled in magnitude so that each fills the gray scale, but with zero always represented by the same gray level. Black depicts off-regions in the model V1 receptive field, white depicts on-regions.

To characterize V1 model receptive fields, feedforward connection weights from on-center type and off-center type LGN cells coding for the same spatial location are summed for each of the model's 128 V1 cells. These summed weights are shown in Figure 2. This gives an indication of the V1 receptive fields, as V1 responses in the model are linear across their on and off inputs (Methods, equation 7). After training, the receptive fields show orientation tuning as found for simple cells in V1.

**Figure 3:**
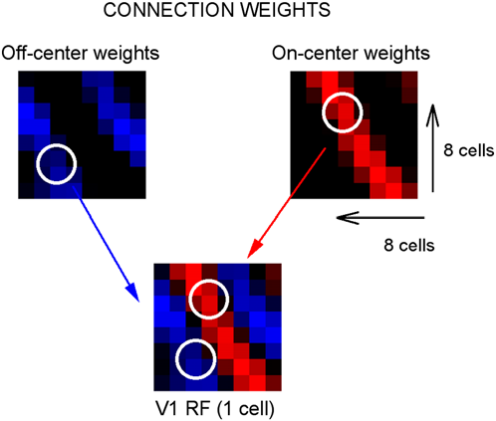
Connection weights after training. The figure depicts learned connection weights from 64 LGN off-center type units and from 64 LGN on-center type units to one representative V1 unit. Red: connection weights from on-center type cells, blue: connection weights from off-center type cells. Brighter values indicate higher connection weights. The value zero is represented by the color black. The on- and off-center units are spatially aligned with the on- and off-zones of the model V1 receptive field.

In Figure 3, the relation between the learned receptive fields in model V1 and the properties of LGN units is further investigated. The figure depicts the connection weights from on-center type cells to a given V1 model neuron, as well as those from off-center type cells to the same V1 neuron. The on- and off-center units are spatially aligned with the on- and off-zones of the model V1 receptive field, as first proposed by Hubel and Wiesel [27] and later confirmed experimentally [22], [28]–[30]. Similar results are found for the connection structure of other V1 model neurons (results not shown). Note that this alignment is not predetermined in the model. The connections are initially random and are adjusted as a consequence of the model's learning rule together with exposure to natural images.

### Reversal of polarity due to predictive feedback

**Figure 4:**
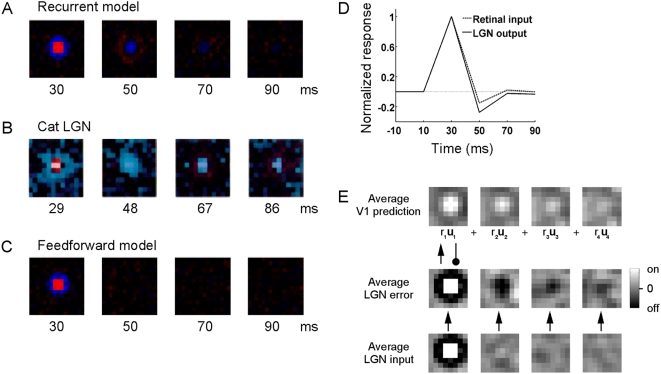
Spatio-temporal response of LGN on-center type cell. The response was mapped with the reverse correlation algorithm, using either non-biphasic retinal inputs (A–C,E) or biphasic retinal inputs (D). (A) Spatio-temporal response of an on-center type cell in model LGN. Responses were obtained by cross-correlating stimulus and response at the time intervals given below the figures. Red: response to bright stimulus at that location, blue: response to dark stimulus at that location. Note the change in sign after 50 milliseconds. Similar results were obtained for other LGN on-center type units. (B) Spatio-temporal response profile of on-center type cells in cat LGN obtained with the reverse correlation algorithm [1]. (C) The removal of feedback in the model causes the previously biphasic responses to disappear. (D) Temporal response profile of on-center type cell in a model with biphasic retinal inputs. Model activity is normalized by the initial response magnitudes. The biphasic response in LGN is more pronounced in the presence of predictive feedback compared to a situation in which the LGN response is fully determined by biphasic retinal input (for comparison, see e.g. [21]). (E) Average model LGN and V1 representations after reference stimuli consisting of bright stimulus regions have been presented. Black depicts off regions, white depicts on regions. When V1 predictions of the bright reference stimulus arrive in model LGN, they are compared against a new and unexpected stimulus representation. The difference between the predicted bright region and the second stimulus is negative, exciting LGN off-center type cells.

To ascertain whether biphasic responses can be interpreted as the result of predictive feedback, we first consider a model with non-biphasic inputs (Fig. 4A and 4C). The spatio-temporal response of model on-center type geniculate cells is calculated using a reverse correlation algorithm (Methods, see also [1],[2],[31]). The time course of the model response is shown by a series of receptive field maps calculated for different delays between stimulus and response in Figure 4A. For comparison Figure 4B shows results obtained from on-center type cells in cat LGN [1]. As in cat LGN, model on-center type receptive fields are arranged in center and surround, and the bright-excitatory phase is followed by a dark-excitatory phase. Removing feedback in the model causes the previously biphasic responses to disappear (Fig. 4C), supporting the idea that predictive feedback may be important for rebound effects in neural response profiles. To determine whether predictive feedback can result in geniculate biphasic responses stronger than those in the retina, the model is modified to simulate biphasic retinal inputs (Methods). The temporal response profile of model on-center type cells is obtained using reverse correlation and illustrated in Figure 4D. Predictive feedback interactions cause reversals of polarity in LGN to be more pronounced than the retinal input, as has been observed in physiology [21].

Why do biphasic responses appear in the mapped model LGN receptive fields? Recall that reverse correlation uses a large number of white noise stimuli presented in rapid succession, resulting in visual changes much faster than most natural input the system would encode. Consider when a stimulus consisting primarily of bright regions is presented to the model (Fig. 4E). On-center type LGN cells will respond to the onset of this stimulus. On zones in the LGN are linked to on zones of receptive fields in V1, which soon start to increase activation and make predictions. However, by the time that predictions of the first stimulus arrive in lower-level areas, the initial representation of the bright stimulus has been replaced by a second white noise stimulus, and the prediction is compared against a new and unexpected stimulus representation. Any given second white-noise stimulus region can be of either high or low luminance; however, the running average luminance will lie in between. In reverse correlation, predictive processing shows up as a comparison against this running average white-noise stimulation. The predicted bright region is of higher luminance than the average second stimulus, causing off-center type cells to respond to the offset of the bright reference stimulus.

Reversals in polarity of model LGN cells are most profound in a small time window after presentation of the reference stimulus but disappear gradually later on. This happens because the initial prediction is dynamically updated to include predictions of stimuli presented after the reference stimulus, bringing new predictions closer to the average white-noise stimulation. Note that reversals in polarity will appear as long as predictions deviate from the average white-noise stimulation; the precise amount of overlap between prediction and stimulus is not critical.

**Figure 5:**
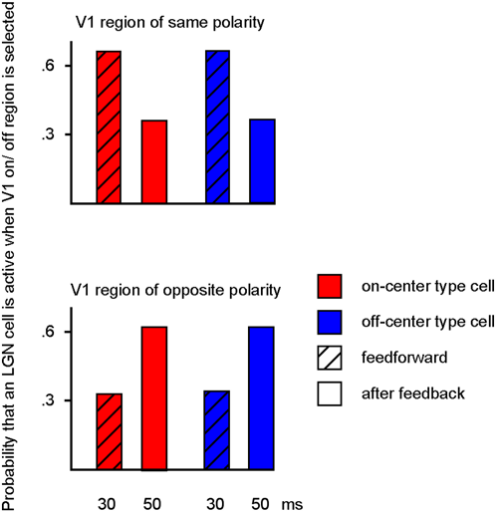
Effects of feedback on LGN on-center and off-center type cells. Dashed: probability that the LGN cell coding for this location is active (i.e. response>0) in the first feedforward sweep of the model when a V1 region will subsequently be selected that codes for the same or opposite polarity, blank: probability that the LGN cell is active after the first feedforward-feedback pass (i.e. when feedback exerts its effect) when a V1 region is selected that codes for the same or opposite polarity. Red: on-center type cell, blue: off-center type cell. The results were obtained after presenting the model with a white-noise stimulus every 100 milliseconds for a total of 10,000 images. Comparing initial feedforward activity with subsequent LGN activity shows that feedback has a negative influence on cells of similar sign, and a positive influence on cells of opposite sign. Thus, the probability that an LGN off-cell is active increases after feedback from a V1 on-region (lower right, blue), and the probability that an LGN on-type cell is active decreases after feedback from an on-region in V1 (upper left, red).

These findings suggest a specific pattern of influence of feedback on LGN cells, in which the simple cell off-zones mediate inhibitory influences to off-center LGN cells and excitatory influences to on-center LGN cells. This effect is further investigated and quantified in Figure 5. For all model on- or off-center LGN receptive fields that are aligned over a V1 receptive field region of the same polarity, firing rates decrease due to feedback (Fig. 5, top). Where the overlapping fields are of reversed polarity, there is an increase in firing rate (Fig. 5, bottom). This effect is consistent with recent results from neurophysiology showing that the influence of V1 simple cells on LGN on- and off-cells is phase-reversed [22], and further corroborates the hypothesis that predictive feedback is important in mediating responses of LGN cells.

## Discussion

We have shown that a model that encodes an image using predictive feedforward-feedback cycles can learn the brain's LGN-V1 connectivity structure, in which the structure of V1 receptive fields is linked to the spatial alignment and properties of center-surround cells in the LGN [27],[28],[30]. In addition, the model captures reversals in polarity of neuronal responses in LGN [1]–[3] and a phase-reversed pattern of influence from V1 simple cells on LGN cells [22]. These results corroborate the idea that the visual system uses predictive feedforward-feedback interactions to efficiently encode natural input.

The natural visual world is dominated by low temporal frequencies [32], causing the retinal image to be relatively stable over the periods of time considered in the model. However, under certain conditions visual inputs do change rapidly—more rapidly than most natural inputs the system would encode. One such situation is brought about by reverse correlation mapping, in which a white-noise stimulus is presented at a temporal frequency much higher than the temporal dynamics of natural visual input. In such a case, higher-level predictions of the reference stimulus are compared against a new and unexpected white-noise stimulus, which emerges in the responses of on-center type model cells as a bright-excitatory phase followed by a dark-excitatory phase. We hypothesize that similar predictive coding mechanisms are at play in geniculate and cortical cells whose spatiotemporal response profiles also display reversals in polarity over short periods of time [3],[5],[6],[33].

Geniculate cells receive many more feedback connections (around 30%) than feedforward connections (around 10%) [34]. In addition, it is known from both cat and monkey neurophysiology that feedback signals from primary visual cortex affect the response properties of LGN cells ([35],[36] see for review [34],[37],[38]). For example, feedback from V1 seems to affect the strength of center-surround interactions in LGN ([34],[37],[38] but see [39]). Geniculate cells respond strongly to bars that are roughly the same size as the center of their receptive field, but responses are attenuated or eliminated when the bar extends beyond the receptive field center [35],[40]. Neurons that respond in this way are also known as end-stopped neurons, and this property has been found to depend on feedback signals from primary visual cortex [35],[40].

A previously published model has successfully captured end-stopping and some other modulations due to surround inhibition in terms of predictive feedback [14]. Like here, the predictive feedback model was trained on natural images, in which lines are usually longer rather than shorter, resulting in higher-level receptive fields optimized for representing longer bars. Thus, when presented with shorter bars, the model's higher-level units could not predict their lower-level input, and error responses in the lower-level neurons could not be suppressed. This resulted in more vigorous responses for shorter bars than for longer bars, similar to end-stopping in geniculate neurons [35]. Here, we have extended the predictive feedback framework to also include rebound effects in LGN. Although responses of LGN cells tend to follow many of the characteristics of their strongest retinal inputs [20], biphasic responses are stronger in geniculate neurons than in the retinal neurons driving their response [21], suggesting that the cells may receive further sources of input. Our simulations indicate that these stronger components in the biphasic geniculate response may result from predictive feedback interactions, similar to end-stopping and some other inhibitory effects [34],[37],[38]. Reversals in polarity have also been described for several cortical areas that do not receive direct input from biphasic retinal cells [3]–[6] and that are too complex to result from retinal responses (e.g., for orientation or motion [4],[6]). We hypothesize that these response profiles result from similar mechanisms of predictive feedback. Indeed, neurophysiological studies have ascribed some cortical rebound effects to network interactions [4],[6], and computational work similarly suggests the involvement of cortical projections [41]. Our work extends these studies by providing a computational explanation for these effects.

Previous authors have suggested mechanisms that could account for the stronger biphasic responses in the LGN, such as higher LGN thresholds [42], inhibitory feedback from the perigeniculate nucleus, or feedforward inhibition [20]. In addition, a variety of models has been proposed to account for orientation selectivity in early visual cortex [9], [43]–[45]. However, our model differs from earlier work in that it offers a computational, not a mechanistic, explanation of these early visual response properties [8]. Furthermore, the framework provides a parsimonious explanation for a number of neurophysiological effects. For example, the model not only captures biphasic responses and orientation selectivity, but also a phase-reversed influence of cortical feedback to LGN, as well as end-stopping and some other modulations due to surround inhibition in V1 and LGN [14]. Reversals in polarity have also been described for many areas in cortex [3]–[6]. Consistent with our interpretation, neurophysiological studies have ascribed some of these biphasic responses to network interactions [4],[6]. While a number of mechanisms can be proposed to account for many of these effects individually, the computational explanation proposed here offers a simple, unifying framework in which to understand all of these effects.

While predictive coding could work through local computations between neighboring neurons (providing a possible explanation for biphasic responses in the retina [13],[46]), we argue that it would be computationally advantageous to (also) implement predictive operations through feedback projections. Feedback mechanisms allow the system to remove redundancy and decorrelate visual responses between areas. Moreover, higher-level cortical receptive fields are larger and encode more complex stimuli, allowing for predictions of higher complexity and larger regions in the visual field. A strong prediction of the model would therefore be that biphasic responses are attenuated in the LGN, or absent in cortex, without cortical feedback.

The model uses subtractive feedback to compare higher-level predictions with actual lower-level input. In physiology, this process could be mediated by, for example, local inhibitory neurons in the same-level area together with long-range excitatory connections from the next higher-level area (for a similar connectivity scheme, see e.g. [34]). Here we have shown that these comparisons can result in reduced as well as enhanced lower-level responses. Support for a dependence of some inhibitory and excitatory effects on top-down feedback has been found in neurophysiology [47]–[50].

We have considered only two hierarchical levels but the model could easily be extended to include more cortical areas. In an extended model, each level would have both coding units and difference detecting units (for a concrete example, see Figure 2 in [15]). Coding units would not only predict their lower-level input but also convey the current estimate to the error detectors of the same-level area. Error detectors then signal the difference between their input and its prediction to the next higher level, until finally one prediction becomes dominant in the entire system. The model suggests that more accurate higher-level predictions, or equivalently greater overlap between the visual input and higher-level receptive fields, results in reduced activity of lower-level difference detectors. In contrast, when top-down predictions in the model are off, lower-level difference detectors enhance their responses. Consistent with this, recent fMRI studies have shown that increased activity in higher-level areas accompanies decreased responses in lower-level areas, presumably due to feedback processing [51]–[53]. Other imaging studies have found supporting evidence for predictive feedback as well [54],[55].

The predictive feedback framework suggests that higher-level coding neurons enhance their activity when stimuli are presented that match their receptive field properties (rather than decrease [56]), in accordance with neurophysiology [27], [57]–[59]. Subsequent feedforward-feedback passes refine the initial predictions, until finally the entire system settles on the mostly likely interpretation. Coding an image using recurrent cycles of processing incurs a cost in time, but has the advantage of resolving error signals in even the earliest sensory areas. Moreover, recurrent cycles of processing are less costly in time when the system forms a hierarchy. The most likely predictions are computed first and sent on to higher-level processing areas, which do not have to wait to begin their own computations, enabling initial rapid gist-of-the-scene processing and subsequent feedforward-feedback cycles to fill in the missing details. In accordance with this, psychophysical studies have shown that some global aspects of a stimulus can be detected very rapidly while detailed aspects are reported later in time [60]–[62], and neurophysiological studies have found dynamic changes in tuning properties of both lower-level and higher-level neurons consistent with these ideas ([33],[63],[64] see also [65],[66]).

It is likely that top-down signals serve many computational functions, of which the sparsifying mechanism suggested here is but one. Also, the effect of top-down signals in general is not best described as either inhibitory or excitatory. The effect can be of many different kinds, depending on the specific computational goals the top-down interaction fulfills. For example, it has been proposed that higher-level areas feed anticipatory signals back to earlier areas, enhancing neural responses to a stimulus that would otherwise fall below threshold [67]. This is probably best implemented as an excitatory interaction between higher-level anticipation and the incoming lower-level signal. Feedback could also act as a bayesian style prior [68]–[70], and adapt early level signals according to different sensory or behavioral conditions [71]. In view of these previously suggested roles of feedback, the mechanism presented here should be regarded as a relatively low-level mechanism that automatically creates sparser solutions, rather than the more flexible, higher-level mechanism that sets specific behavioral demands.

In conclusion, rebound effects are a common feature in reverse correlation mapping and have been described in several visual areas. For example, biphasic responses have been found for neurons in LGN [1]–[3], as well as in primary visual cortex [3],[4], and reversals in selectivity in the motion domain have also been found for neurons in MT [5],[6]. Here, we have explained these biphasic sensory responses in terms of predictive feedback. Moreover, we have shown that a model that processes its inputs using predictive feedback can learn the brain's LGN-V1 connectivity structure [27],[28],[30] and captures a phase-reversed pattern of influence of feedback [22]. These results corroborate the idea that predictive feedback is a general principle used by the visual system to efficiently encode its natural inputs.

## Methods

### Model dynamics

Here we briefly discuss model equations and parameters. The interested reader is referred to [25]. The input is obtained from 768 by 768 pixel black-and-white images of natural surroundings (Fig. 2A), filtered with a zero-phase whitening/lowpass filter [7],[9]:
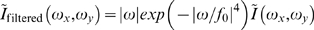
(1)where the tilde represents the fourier transform in 2D, and 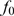 = 300 cycles/image. The initial activation values of on-center type cells are obtained from the filtered images by subtracting the mean and taking positive values:
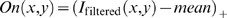
(2)


The initial activation values of off-center type cells are obtained from negative pixel values that are rectified:
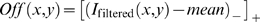
(3)


We limit the LGN input into the model's second layer to 8 by 8 (64) on-center type cells and 8 by 8 (64) off-center type cells. This LGN ‘patch’ is randomly selected from the filtered image and represented as a single vector (128 values), unless described otherwise. At any given time step in the model, either the on-center cell or its off-center counterpart coding for the same spatial location is active.

**Figure 6:**
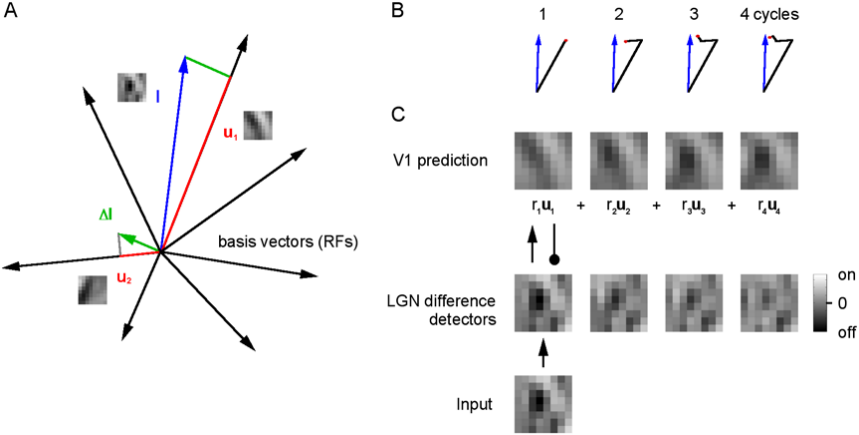
Predictive feedback using the matching pursuit algorithm. (A) Model receptive fields (RFs) are represented as basis vectors. When the input (blue vector) arrives in model V1, a basis vector that has high overlap with the input is selected (red vector 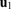). The V1 basis vector weighted by its response is then subtracted from the input and the selection-subtraction process is repeated on the residual LGN representation (green vector). (B,C) Model V1 prediction and residual LGN representation over time. (B) The blue vector represents the actual input, its prediction is represented by the red dot. (C) Black depicts off-regions, white depicts on-regions. A prediction is obtained by summing the selected V1 basis vectors weighted by their response. LGN difference detectors represent the error between V1 prediction and actual input. (B,C) Subsequent feedforward-feedback cycles refine the higher-level prediction of the input. Without predictive feedback, the model would represent just the initial, less accurate prediction.

The second layer, which would correspond to an orientation column in cortical area V1, is represented by 128 units. In the language of the model, the synaptic weights between LGN and V1 units form basis vectors that represent the preferred stimulus of the model V1 neurons (Fig. 6A). Model V1 predicts its LGN input 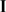 as a linear combination of 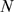 active basis vectors, where the weighting coefficient of each basis vector 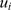 is given by the response 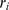 of its corresponding V1 neuron:
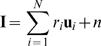
(4)in which 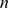 is a stochastic noise process. The 64 on-type and 64 off-type connections are combined to form a single basis vector of 128 values, unless described otherwise. To choose the 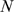 V1 neurons that best predict a given input (i.e., neurons that are active), we use a modified version of the matching pursuit algorithm [72]. Matching-pursuit uses the least number of basis vectors or equivalently the least number of active V1 neurons to accurately predict its input [72]. In a deterministic version of the algorithm, the first vector is chosen as the vector 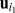 that minimizes
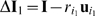
(5)


At the next time step, an additional vector is chosen that minimizes
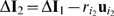
(6)and so on, where the response 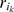 of the vectors is given by
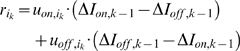
(7)and the vectors are subdivided into 64 on-values and 64 off-values. This deterministic version was modified for the learning algorithm to be optimal in terms of sparseness [25]. Thus, after learning not only do the V1 units make more accurate predictions, but also few of them participate in any given prediction. The modification is as follows: at each time step, a V1 unit is selected randomly from the distribution
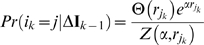
(8)where 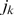 is the index of the 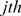 unit in the 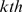 iteration, 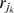 is given by equation (7), 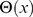 is the Heaviside function, 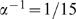 is a temperature parameter and 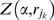 is a normalizing term given by:
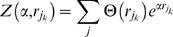
(9)


Thus, the probability with which a unit is selected increases when its receptive field structure better predicts the lower level input and its response is higher. To guarantee optimality, the response of a selected unit 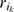 should be drawn from a normal distribution 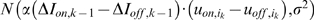 with small variance [25], but the effect of this process is negligible so that in practice the response of a neuron in the modified model is given by equation (7). The selected basis vector weighted by its neuronal response is then subtracted from the input. This subtraction represents the predictive feedback process assumed to take place between V1 and LGN, and is essential to the predictive coding theory: it reduces output redundancy by allowing LGN difference detectors to represent only the error signal, and no longer the predicted components now represented in V1. Furthermore, it optimizes the higher-level predictions (or equivalently, minimizes the prediction error). For a further discussion of the effectiveness of this approach, see [25]. The LGN on-center type cell and LGN off-center type cell code for two opposite sides of the same dimension. Thus, wherever the subtraction process results in negative LGN values, or equivalently when the value crosses the dividing edge between the two dimensional sides, the value is rectified and added to the activation value of the unit's counterpart (i.e. negative value of on-center unit is rectified and added to activation value of off-center unit coding for the same location, and vice versa). The feedforward-feedback cycle is then repeated on the residual input so that after 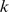 iterations the residual LGN input is given by
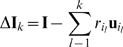
(10)


In words, the number of active V1 neurons increases at each time step in the model, and their combined prediction is subtracted from the actual LGN input. To see how combining active V1 units results in better predictions of the input, consider when a bar of a certain orientation is presented to the model, together with a small dot next to the bar. A likely first prediction could capture the bar, but not necessarily also the dot. However, this initial prediction will be updated in later feedforward-feedback cycles when other V1 neurons become active, so that the neurons will together represent both the bar and the dot. This is what combining V1 neurons represents, a prediction that is updated in each cycle to better capture the V1 input (see also Fig. 6B and 6C). We assume model V1 responses to be stable and non-decaying over the time scales considered. Note that feedback connections in the model follow the alignment of feedforward connections, which has been observed experimentally [22],[23]. We assume delays of 20 milliseconds for predictive feedback effects to set in, which complies with the usual response lag of about 10 milliseconds in the next higher-order visual area [73],[74] together with similar conduction times for feedforward and feedback connections [75]. Although this estimate is likely at the longer end of the range [76], the model does not critically depend on the value of this parameter and similar results would have been obtained using shorter time delays. As the model does not incorporate neural structures earlier than the LGN, we added 30 milliseconds to the data points in the figures to account for the delays before the LGN [73].

### Learning rule

To enhance the sparseness of the neural code and better capture the input statistics, basis vectors are updated in each feedforward-feedback cycle. This is done by minimizing the description length (MDL) or entropy of the joint distribution of inputs and neural responses [25]. MDL chooses as the best model the one that enables the shortest code length for both prediction error and model parameters in bits (in base 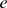) [77],[78]. As a consequence, it favors accurate, yet sparse, neural representations. However, the same learning rule can also be obtained from the gradient of the error function for the 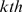 feedforward-feedback cycle [25]:
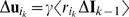
(11)where 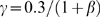 is the learning rate, in which 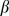 is initially equal to 1 and increases by 1 every 1000 image patches. V1 basis vectors are normalized, and initialized using 64 random values with zero mean: positive values are taken as the initial values of the entries coding for on-type inputs, negative values are rectified and taken as the initial values of the off-type entries of the basis vector, the remaining 64 entries are initialized with value zero. Initializing all 128 entries of the basis vector with random values gives similar results. Because neuronal receptive field properties might be largely determined by the statistics of their natural input [7],[11],[14],[16], the basis vectors are trained on 10,000 image patches extracted from 16 natural scenes. The model receptive fields are trained using static natural images (for receptive fields obtained from time-varying natural inputs see [45], and see [15] for a description within the predictive coding framework). The model is allowed to process each image for four feedforward-feedback cycles, which corresponds to around 100 milliseconds (see above). Parameter values are kept constant throughout all simulations. While natural input is not expected to be completely static, even over the short time scales considered here, we argue that this is not highly relevant to the general results of the simulation. The critical factor for our results is that reverse correlation stimuli are presented on a time scale much faster than the system's typical inputs. The temporal dynamics used in reverse correlation mapping are, indeed, much faster than most natural inputs, as the natural visual world is dominated by low temporal frequencies [32]. Including time-varying V1 receptive fields and/or training the system on natural dynamical images presented for seconds would therefore result in similar biphasic responses, given natural dynamics that are slower than the temporal dynamics of reverse correlation. For example, Kiebel et al. [16] use a predictive feedback framework to model temporal receptive fields, resulting in similar lower-level error signals when the actual input deviates from the expected temporal dynamics.

### Interpretation of model activation values

To model the spatiotemporal response of LGN neurons, we have to relate model scalar activation values directly to biology. Several possibilities exist; we emphasize, however, that the model does not explicitly implement any of these interpretations and is, in fact, very general. One possibility is that activation values of model units stand for the mean firing rate of a group of functionally similar neurons in physiology [79]–[82]. This interpretation is corroborated by most neurophysiological studies that show a correlation between increased firing rates and behavioral measures. The model is also compatible with the idea that neurons code information in the precise timing of their spikes. This view has received increasing attention over the past years [83]–[85] as more data is becoming available suggesting that spike timing may be important for neural communication [86]–[89]. Specifically, scalar activation values in the model can be interpreted as indicating the time from spike arrival to a reference signal, taking this small delay in time between a single spike and the reference as the information carrier [85],[90]. Direct neurophysiological evidence for this signaling strategy has been obtained in hippocampus [91],[92] and in human somatosensory system [93]. Here, we interpret the model's activation values along the spike timing lines and take scalar activation values of model units as information transmitted using one spike. We do not implement the reference signal explicitly but argue that the model could easily be amplified to take this into account. We emphasize, however, that we obtain similar results if we interpret the model's activation values as firing rate (i.e. indicating a number of spikes).

### Reverse correlation

The spatio-temporal response of on-center type units in model LGN (Fig. 4) was calculated using a reverse correlation algorithm ([1],[2],[31] see also C.-I. Yeh et al., Soc. Neurosci. Abstr. 163.21, 2008). This algorithm presents neurons with an image sequence of white-noise stimuli (where each pixel is either maximally black or white with equal probability), records the stimuli that precede a response (i.e. the activity value of the recorded model neuron is above zero) by a fixed time interval, and then averages across the recorded stimuli; the receptive field is defined as this average stimulus that preceded the response by the given time interval. Following [1], the model was presented with a new white-noise stimulus every 20 milliseconds for a total of 50,000 presentations. We used time intervals between response and stimulus of 30, 50, 70 and 90 milliseconds (see also Fig. 4A), and obtained a composite receptive field by taking the difference of responses to bright stimuli and dark stimuli [2]. We used a spike timing interpretation of model responses and recorded stimuli when activation values in the model were above zero. However, we obtain similar results if we interpret model activation values as firing rates and weigh recorded stimuli by the unit's activation value.

### Biphasic retinal input

In the modified model, the initial feedforward input into on-center type LGN cells is obtained using equation 2, and then this bottom-up input is updated in each feedforward-feedback cycle as follows:
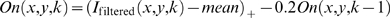
(12)where 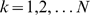 is the number of feedforward-feedback cycles in the model. The retinal input into off-center type LGN cells is initialized using equation 3, and then updated in a similar fashion in each feedforward-feedback cycle. When mapped with reversed correlation, this results in retinal input that is biphasic in structure (Fig. 4D).
